# Polyautoimmunity in patients with cutaneous lupus erythematosus: A nationwide sex- and age-matched cohort study from Denmark

**DOI:** 10.1016/j.jdin.2023.07.018

**Published:** 2023-08-25

**Authors:** Christoffer S. Graven-Nielsen, Ida.V. Vittrup, Anna J. Kragh, Fredrik Lund, Sofie Bliddal, Kristian Kofoed, Salome Kristensen, Allan Stensballe, Claus H. Nielsen, Ulla Feldt-Rasmussen, René Cordtz, Lene Dreyer

**Affiliations:** a Center for Rheumatology Research Aalborg (CERRA), Aalborg University Hospital, Aalborg, Denmark; b Department of Medical Endocrinology and Metabolism, Copenhagen University Hospital (Rigshospitalet), Copenhagen, Denmark; c Institute of Inflammation Research, Center for Rheumatology and Spine Diseases, Copenhagen University Hospital (Rigshospitalet), Copenhagen, Denmark; d Skin Clinic Rødovre, Rødovre, Denmark; e Department of Clinical Medicine, Aalborg University, Denmark; f Department of Health Science and Technology, Aalborg University, Denmark; g Clinical Cancer Research Center, Aalborg University Hospital, Aalborg, Denmark; h Department of Clinical Medicine, Faculty of Health and Medical Sciences, University of Copenhagen, Copenhagen, Denmark

**Keywords:** autoimmune diseases, cutaneous lupus erythematosus, epidemiology, polyautoimmunity, thyroid autoimmunity

## Abstract

**Background:**

Polyautoimmunity is defined as having 2 or more autoimmune diseases. Little is known about polyautoimmunity in patients with cutaneous lupus erythematosus (CLE).

**Objectives:**

To estimate prevalence and 5-year incidence of non–lupus erythematosus (LE) autoimmune diseases in patients with CLE.

**Methods:**

Patients with CLE were identified In the Danish National Patient Registry and each patient was age- and sex-matched with 10 general population controls. Outcome information on non-LE autoimmune diseases was obtained by register-linkage between Danish National Patient Registry and the National Prescription Register. The risk ratio (RR) for prevalent non-LE autoimmune disease at time of CLE diagnosis was calculated in modified Poisson regression; and hazard ratios (HRs) for incident non-LE autoimmune disease were estimated in Cox regression analyses.

**Results:**

Overall, 1674 patients with CLE had a higher prevalence of a non-LE autoimmune disease than the comparators (18.5 vs 7.9%; RR 2.4; 95% CI, 2.1 to 2.6). Correspondingly, the cumulative incidence of a non-LE autoimmune disease during 5 years of follow-up was increased for the patients with CLE: HR 3.5 (95% CI, 3.0 to 4.0).

**Limitations:**

Risk of detection and misclassification bias, mainly pertaining to the CLE group.

**Conclusion:**

Patients with CLE had higher prevalence and 5-year cumulative incidence of a non-LE autoimmune disease than the general population.

## Introduction

Cutaneous lupus erythematosus (CLE) is an autoimmune, cutaneous disease characterized by well-defined skin lesions, divided into subtypes acute CLE, subacute lupus erythematosus (SCLE), and discoid lupus erythemat, often located in areas exposed to sunlight; and ultraviolet light can induce both onset and relapse of the disease.^1^^,^^2^

In Denmark, the annual incidence of CLE is 2.7 per 100,000 person years with a 4:1 female predominance.^3^ The prevalence of CLE in Denmark is unknown, but a study from Minnesota, United States of America, with a predominantly Caucasian population found a prevalence of 73.3 per 100,000 persons.^4^^,^^5^

A phenomenon in autoimmunity is that development of another autoimmune disease in a patient with a preexisting autoimmune disease is more likely than development of a first autoimmune disease in another individual.^6^ The underlying reasons are not fully understood but could be due to similar immunopathological mechanisms and genetic factors in many autoimmune diseases. A patient with 2 or more autoimmune diseases is said to have polyautoimmunity.^7^^,^^8^ Little is known about the prevalence and incidence of polyautoimmunity in patients with CLE compared with controls in the general population in a nationwide study.^9^

Thus, the aim of this study was to estimate the prevalence of non–lupus erythematosus (LE) autoimmune disease at the time of CLE diagnosis and the subsequent 5-year incidence of non-LE autoimmune disease compared with matched controls from the general population.

## Methods

### Study design

This nationwide register-based Danish cohort study investigates prevalence and 5-year incidence of polyautoimmunity among recently diagnosed patients with CLE compared with the prevalence and 5-year incidence of autoimmune disease in age- and sex-matched controls from the general population. The study period was 1998-2021.

### Data sources

#### The Danish Civil Registration System

By use of the Central Person Register-number, assigned to all Danish residents, the Danish Civil Registration System (CRS) allows for linkage between all nationwide registers.^9^ For each Danish resident, the CRS contains personal information, such as gender, date of birth, and if relevant, dates of migration and death. The CRS can be used for sampling of comparator cohorts in studies.

#### The Danish National Patient Register

The Danish National Patient Register (DNPR) is a nationwide administrative register that contains standardized data from all hospitals in Denmark since 1978.^10^ All public hospitals in Denmark are required by law to submit data on admissions, diagnosis code(s), and treatment(s) for every patient. Thus, when a patient is in contact with a hospital, a contact is recorded in the DNPR with 1 or more International Statistical Classification of Diseases, Tenth Revision (ICD-10).

#### The Danish National Prescription Register

The Danish National Prescription Register covers the Danish population and includes data on all reimbursed prescriptions redeemed at community pharmacies. We gained information on date of redemption, and Anatomical Therapeutic Chemical Classification System code.^11^

### Study population

To identify patients with CLE, all first-time diagnoses of CLE (ICD-10 codes L93.0-L93.2) in patients aged ≥18 years were identified in the DNPR from 1998 to the end of 2020. The CLE diagnosis should originate from a department of dermatology to reduce misclassification bias. Patients with a DNPR record of systemic lupus erythematosus (SLE) before their CLE diagnosis were not included. The CRS was used to sample a control group from the general population of 10 individuals matched on year of birth and sex to each patient with CLE (see Supplementary Fig S1, available via Mendeley at https://data.mendeley.com/datasets/h2bvck3x5s/1). Matched controls with SLE were excluded. For the case patient with CLE and the matched individuals of that case patient, the index date from which all were followed was the date of the CLE diagnosis.

Patients with CLE and matched controls that were later registered with a diagnosis of SLE were followed up in their respective groups until the date of their SLE diagnosis at which point they were censored. SLE was not part of the composite outcome.

### Outcome

The primary outcome was defined as the presence of 1 or more non-LE and non-SLE autoimmune disease(s). A list of the specific autoimmune conditions included in the composite outcome and their register-specific definition is available in Supplementary Table S1 (available via Mendeley at https://data.mendeley.com/datasets/h2bvck3x5s/1).

### Statistical methods

The prevalence of autoimmune disease was calculated as the proportion of patients with an autoimmune diagnosis other than CLE registered before the index date, and for matched controls as any non-LE autoimmune disease before the index date. Modified Poisson regression was used to calculate the risk ratio (RR) with 95% confidence intervals (95% CI) for prevalent autoimmune disease in patients with CLE with matched controls as reference.

The age and sex-standardized incidence rate with 95% CI of developing another autoimmune disease in the subsequent 5 years was calculated. A cause-specific Cox regression with death as a competing risk was used to calculate hazard ratios (HRs) for developing an autoimmune disease in the 5 years after the index date with matched controls as reference group. Subjects were censored at time of an event, that is, any incident autoimmune disease, at the end of the 5-year follow-up period, emigration, or death, whichever occurred first.

The Aalen-Johansen estimator was used to calculate the 5-year cumulative incidence (in %) of autoimmune disease while taking into account the competing risk of death.^12^

Data management and all analyses were performed in R version 3.6.1.^13^ The level of statistical significance was set at 5%.

### Sensitivity analyses

To test the robustness of the outcome definition, an analysis was performed in which all rheumatic autoimmune conditions and autoimmune diseases that could represent undiagnosed or progression to SLE were excluded from the composite outcome (see Supplementary Table S1). This analysis was performed to investigate if any potential difference in prevalence and incidence of autoimmune diseases between patients with CLE and matched controls remained when excluding conditions that could in fact represent overlap conditions with CLE/SLE rather than distinct autoimmune diseases.

## Results

### Demographic and clinical characteristics of study population

A total of 1674 patients with CLE were identified from 1998 to the end of 2020. The prevalence and 5-year incidence of non-LE autoimmune diseases in discoid LE (ICD-10 L93.0), subacute CLE (ICD-10 L93.1), and other local LE (ICD-10 L93.2) were calculated, respectively. The baseline demographic and clinical characteristics of the participants are presented in Table I. The median age of the patients with CLE and matched controls was 54 years (IQR 42 to 67 years) at diagnosis and 78.2% were women.

**Table I:** Characteristics of patients with cutaneous lupus erythematosus at time of diagnosis and of age- and sex-matched controls

Variable	Discoid lupus	Subacute lupus	Other local lupus	Cutaneous lupus erythematosus (total)	Matched controls
Number of patients	1002	458	214	1674	16,707
Median age (IQR), y	51.1 (39.4, 63.9)	62.7 (49.3, 71.7)	51.5 (39.2, 65.2)	54.3 (41.7, 66.9)	54.2 (41.6, 67.0)
Women, *N* (%)	768 (76.6)	372 (81.2)	169 (79.0)	1309 (78.2)	13,068 (78.2)
Comorbidities					
Cardiovascular disease, *N* (%)	111 (11.1)	74 (16.2)	28 (13.1)	213 (12.7)	1409 (8.4)
Chronic obstructive pulmonary disease, *N* (%)	32 (3.2)	21 (4.6)	9 (4.2)	62 (3.7)	367 (2.2)
Medication					
Hydroxychloroquine, *N* (%)	149 (14.9)	45 (9.8)	21 (9.8)	215 (12.8)	24 (0.1)
Methotrexate, *N* (%)	16 (1.6)	11 (2.4)	7 (3.3)	34 (2.0)	106 (0.6)
Azathioprine, *N* (%)	24 (2.4)	15 (3.3)	5 (2.3)	44 (2.6)	23 (0.1)
Prednisolone, *N* (%)	113 (11.3)	103 (22.5)	25 (11.7)	241 (14.4)	339 (2.0)
Topical glucocorticoid, *N* (%)	510 (50.9)	271 (59.2)	89 (41.6)	870 (52.0)	1154 (6.9)
Calcineurin inhibitor, *N* (%)	27 (2.7)	11 (2.4)	6 (2.8)	44 (2.6)	43 (0.3)

*IQR*, Interquartile range.

In the CLE group, 18.5% had a non-LE autoimmune condition (and thus polyautoimmunity) at the time of diagnosis compared to 7.9% with autoimmune disease among matched controls resulting in a RR of 1.8 (95% CI, 1.7 to 2.0), see Table II. The most prevalent autoimmune condition at the time of diagnosis in both groups was autoimmune thyroid disease with 6.4% in the CLE group compared with 4.6% in matched controls. Rheumatoid arthritis was the second most prevalent autoimmune disease. The prevalence of polyautoimmunity was only 0.8% in the matched controls resulting in an RR of 22.4 (18.5 to 27.2) for polyautoimmunity in patients with CLE compared to the former. Among the 1674 patients with CLE, 69 (4.12%) had 2 or more autoimmune diseases at time of diagnose compared to 138 of 16,707 (0.83%) of the controls. The corresponding figures for 3 or more autoimmune diseases were 15 (0.90%) and 15 (0.09%), respectively (data not shown). The prevalences of polyautoimmunity in CLE-subtypes discoid LE, SCLE, and other local LE at the time of diagnosis were 15.9%, 23.4%, and 20.6%, respectively, see Supplementary Table S2 (available via Mendeley at https://data.mendeley.com/datasets/h2bvck3x5s/1).

**Table II:** Prevalence of autoimmune diseases in patients with cutaneous lupus erythematosus and matched controls at time of diagnosis and matching, respectively

Variable	CLE (*N* = 1674)	Matched controls (*N* = 16,707)
Autoimmune disease, *N* (%)	310 (18.5)	1319 (7.9)
Polyautoimmunity, *N* (%)	310 (18.5)	138 (0.8)
Type 1 diabetes mellitus, *N* (%)	6 (0.4)	59 (0.4)
Autoimmune thyroid disease, *N* (%)	107 (6.4)	773 (4.6)
Primary adrenocortical insufficiency, *N* (%)	≤3	6 (0.0)
Inflammatory bowel disease, *N* (%)	27 (1.6)	157 (0.9)
Primary biliary cirrhosis, *N* (%)	≤3	8 (0.0)
Autoimmune hepatitis, *N* (%)	9 (0.5)	7 (0.0)
Autoimmune pancreatitis, *N* (%)	0 (0.0)	0 (0.0)
Celiac disease, *N* (%)	5 (0.3)	24 (0.1)
Multiple sclerosis, *N* (%)	10 (0.6)	74 (0.4)
Neuromyelitis optica, *N* (%)	0 (0.0)	≤3
Guillain-Barré disease, *N* (%)	≤3	≤3
Myasthenia gravis, *N* (%)	0 (0.0)	4 (0.0)
Pemphigus vulgaris, *N* (%)	≤3	0 (0.0)
Bullous pemphigoid, *N* (%)	≤3	≤3
Dermatitis herpetiformis, *N* (%)	≤3	≤3
Vitiligo, *N* (%)	6 (0.4)	4 (0.0)
Lichen sclerosus, *N* (%)	4 (0.2)	27 (0.2)
Pernicious anemia, *N* (%)	4 (0.2)	6 (0.0)
Other autoimmune hemolytic anemia, *N* (%)	≤3	5 (0.0)
Iridocyclitis, *N* (%)	≤3	0 (0.0)
Rheumatoid arthritis, *N* (%)	68 (4.1)	189 (1.1)
Eosinophilic granulomatosis with polyangitis, *N* (%)	0 (0.0)	0 (0.0)
Granulomatosis with polyangitis, *N* (%)	≤3	≤3
Myositis, *N* (%)	17 (1.0)	≤3
Scleroderma, *N* (%)	18 (1.1)	6 (0.0)
Sjogren’s syndrome, *N* (%)	68 (4.1)	29 (0.2)
RR (95% CI) of autoimmune disease	2.4 (2.1 to 2.6)	1 (Ref.)
RR (95% CI) of polyautoimmunity	22.4 (18.5 to 27.2)	1 (Ref.)

*95% CI*, 95% Confidence interval; *CLE*, cutaneous lupus erythematosus; *RR*, risk ratio.

When excluding autoimmune rheumatic and hematologic conditions that could be related to CLE or progression to SLE, the prevalence estimates for autoimmune disease decreased to 9.6% and 6.3% in patients with CLE and matched controls, respectively, and the RR decreased to 1.4 (1.2 to 1.5), see Supplementary Table S3 (available via Mendeley at https://data.mendeley.com/datasets/h2bvck3x5s/1). When defining type 1 diabetes mellitus and autoimmune thyroid disease strictly by use of their respective ICD-10 codes, the RR for prevalent autoimmune disease was 2.4 (2.1 to 2.6), Table II.

The 5-year age- and sex-standardized incidence rates (per 1000 person years) of non-LE autoimmune disease were 31.1 (27.1 to 35.7) among patients with CLE and 9.2 (8.5 to 9.9) in the control group (Table III). The corresponding HR in a cause-specific Cox regression was 3.5 (3.0 to 4.0). Incidence rates (per 1000 person years) of non-LE autoimmune diseases in CLE-subtypes discoid LE, SCLE and other local LE were 28.8 (24 to 34.6), 37.5 (29 to 48.5) and 25.9 (17.2 to 39.1) respectively, see Supplementary Table S8 (available via Mendeley at https://data.mendeley.com/datasets/h2bvck3x5s/1). The most frequent diagnoses in the CLE group during the 5-year follow-up period were Sjogren’s syndrome (5.0%) and rheumatoid arthritis (2.5%), whereas autoimmune thyroid disease was the most frequent diagnosis in matched controls (0.9%). The HR for polyautoimmunity was 30.2 (22.5 to 40.7) for patients with CLE compared with matched controls.

**Table III:** 5-year cumulative incidence (*N* and %), age and sex-standardized incidence rate, and hazard ratio for developing an autoimmune disease after cutaneous lupus erythematosus diagnosis

Variable	CLE	Matched controls
Number of patients	1667	16,633
Sum of person years	6713	72,119
Mean follow-up in years, SD	4.3 (1.3)	4.0 (1.6)
Autoimmune disease, *N* (%)	5.4%	2.2%
Incident polyautoimmunity, *N* (%)	5.4%	0.1%
Died, *N* (%)	10.7%	6.2%
Type 1 diabetes mellitus, *N* (%)	0 (0.0)	≤3
Autoimmune thyroid disease, *N* (%)	25 (1.5)	204 (1.2)
Primary adrenocortical insufficiency, *N* (%)	≤3	7 (0.0)
Inflammatory bowel disease, *N* (%)	15 (0.9)	106 (0.6)
Primary biliary cirrhosis, *N* (%)	4 (0.2)	12 (0.1)
Autoimmune hepatitis, *N* (%)	5 (0.3)	11 (0.1)
Autoimmune pancreatitis, *N* (%)	0 (0.0)	≤3
Celiac disease, *N* (%)	4 (0.2)	11 (0.1)
Multiple sclerosis, *N* (%)	≤3	27 (0.2)
Neuromyelitis optica, *N* (%)	0 (0.0)	0 (0.0)
Guillain-Barré disease, *N* (%)	0 (0.0)	9 (0.1)
Myasthenia gravis, *N* (%)	0 (0.0)	≤3
Pemphigus vulgaris, *N* (%)	≤3	≤3
Bullous pemphigoid, *N* (%)	≤3	4 (0.0)
Dermatitis herpetiformis, *N* (%)	0 (0.0)	≤3
Vitiligo, *N* (%)	≤3	6 (0.0)
Lichen sclerosus, *N* (%)	8 (0.5)	48 (0.3)
Pernicious anemia, *N* (%)	≤3	6 (0.0)
Other autoimmune hemolytic anemia, *N* (%)	≤3	5 (0.0)
Iridocyclitis, *N* (%)	0 (0.0)	0 (0.0)
Rheumatoid arthritis, *N* (%)	42 (2.5)	144 (0.9)
Eosinophilic granulomatosis with polyangitis, *N* (%)	0 (0.0)	≤3
Granulomatosis with polyangitis, *N* (%)	≤3	7 (0.0)
Myositis, *N* (%)	11 (0.7)	7 (0.0)
Scleroderma, *N* (%)	8 (0.5)	11 (0.1)
Sjogren’s syndrome, *N* (%)	83 (5.0)	31 (0.2)
Incidence rate of autoimmune disease per 1000 person years	31.1 (27.1 to 35.7)	9.2 (8.5 to 9.9)
HR (95% CI) of autoimmune disease	3.5 (3.0 to 4.0)	1 (Ref.)
HR (95% CI) of incident polyautoimmunity	30.2 (22.5 to 40.7)	1 (Ref.)

*95% CI*, 95% Confidence interval; *CLE*, cutaneous lupus erythematosus; *HR*, hazard ratio.

When excluding the autoimmune conditions that could be related to CLE or (progression to) SLE, the incidence rates decreased more markedly in the CLE group; and were 13.3 (10.8 to 16.3) and 7.4 (6.8 to 8) in patients with CLE and matched controls, respectively (Supplementary Table S4, available via Mendeley at https://data.mendeley.com/datasets/h2bvck3x5s/1). Consequently, the HR also decreased to 1.8 (1.5 to 2.3). Altering the definition of type 1 diabetes mellitus and autoimmune thyroid disease increased the incidence rates proportionally in both groups resulting in an HR of 3.5 (3.0 to 4.0) (Table III).

**Fig 1:**
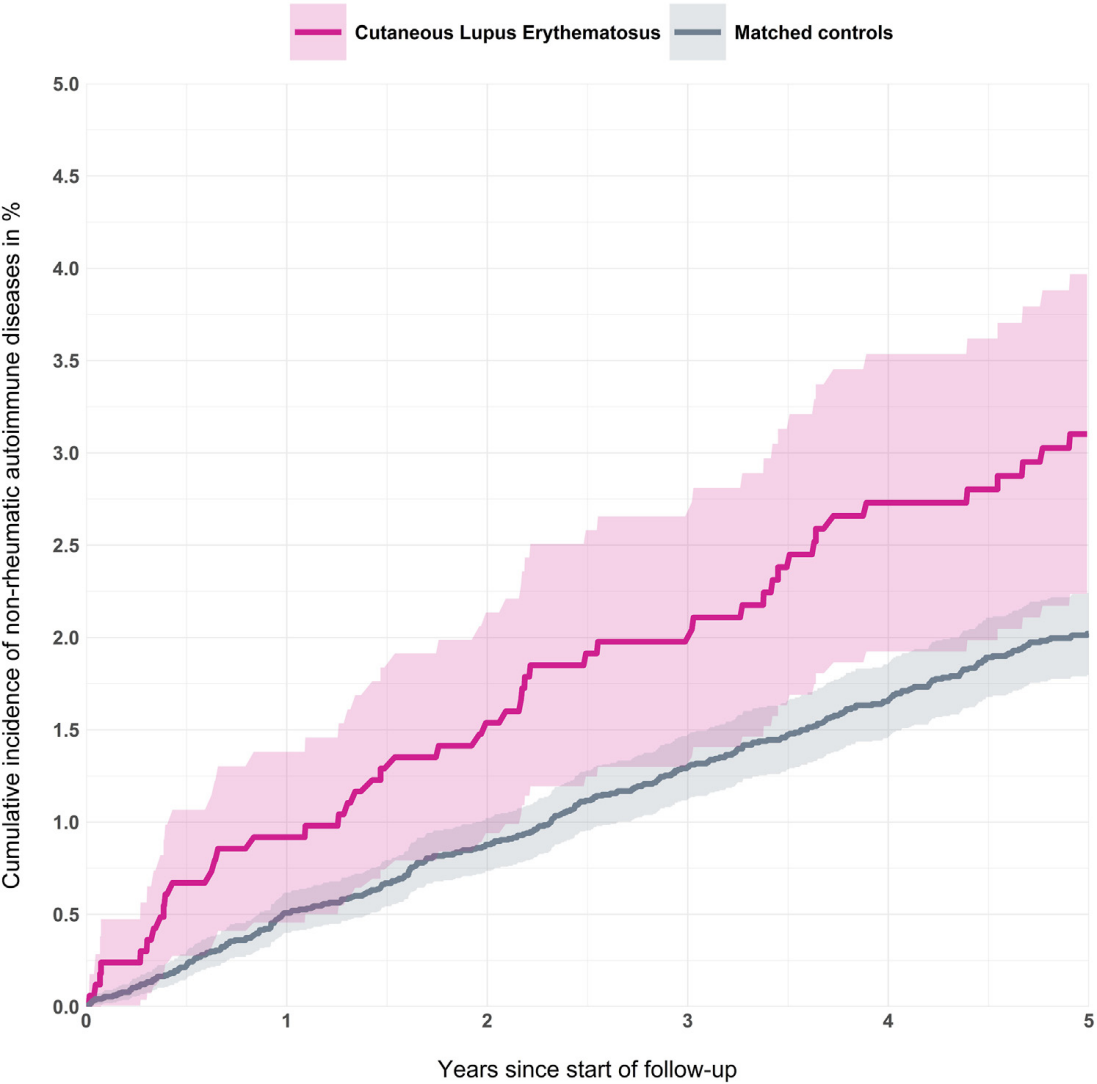
The 5-year cumulative incidence of being diagnosed with an additional autoimmune disease in patients with cutaneous lupus erythematosus and matched controls based on the Aalen-Johansen estimator.

The 5-year cumulative incidence of an autoimmune disease in patients with CLE and matched controls is illustrated in Fig 1 using the Aalen-Johansen estimator. The incidence of autoimmune disease was higher in the CLE group throughout all the 5 years, and at 5 years it was 5.4% compared with 2.2% in matched controls.

The 5-year mortality was higher in the group of patients with CLE at 9.7% compared with 5.6% in the matched controls.

## Discussion

In this Danish nationwide study, the prevalence and 5-year incidence of autoimmune disease were higher in patients with CLE than in matched controls; and the difference between the 2 groups was even more pronounced with respect to polyautoimmunity. Autoimmune thyroid disease was the most frequent autoimmune disease in both groups, whereas Sjogren’s disease and rheumatoid arthritis were the most frequent autoimmune diseases in the 5 years after CLE diagnosis. The prevalence and incidence of polyautoimmunity in CLE-subtypes discoid LE, SCLE, and other local LE were almost equal. The high prevalence of autoimmune thyroid disease seen in patients with polyautoimmunity are supported by other studies,^14^ in keeping with thyroid autoimmunity being by far the most frequent autoimmune phenomenon.

There are multiple potential explanations for our finding of increased autoimmune conditions in the CLE group: a higher degree of immunological dysregulation, genetic predisposition, and higher exposure to certain environmental factors, such as smoking, compared to the matched controls. Smoking is a trigger of CLE.^15^ The higher prevalence of chronic obstructive pulmonary disease in patients with CLE in our study compared with the controls indicates a higher cumulative exposure to tobacco smoking. However, a clear association between cigarette smoking and higher autoantibody concentrations in patients with SLE, and thus possibly also in CLE, has not been consistently demonstrated.^16^^,^^17^ Other autoantibodies are often present in patients with CLE, including antinuclear antibodies, and mainly anti-Ro/SS-A and La/SS-B.^15^ A study investigating the prevalence and distribution of antibodies in 16 autoimmune diseases found anti-Ro/SS-A antibodies to be more prevalent and in higher concentrations in patients with Sjogren’s syndrome, SLE, antiphospholipid syndrome linked to SLE, and primary biliary cirrhosis, than in healthy matched controls.^18^ However, neither anti-Ro/SS-A or anti-La/SS-B antibodies were more prevalent in autoimmune thyroid disease which was the most frequent autoimmune condition in CLE in the present study.

An autoantigen that is highly expressed in the epidermis of skin biopsies from patients with CLE is High Mobility Group Box 1 (HMGB1).^15^ Excessive levels of HMGB1 can be seen in patients with rheumatoid arthritis, whereas antibodies against HMGB1 can be seen in patients with Sjogren’s syndrome.^19^^,^^20^ Interestingly, these are the 2 diseases with the highest incidence following CLE diagnosis in the present study.

In agreement with our results, a cross-sectional study from 2018 and a cohort study from 2019 found that autoimmune thyroid disease was the most frequent autoimmune disease among patients with CLE.^9^^,^^21^ The incidence of various autoimmune diseases in CLE and controls depends on the age-specific incidence rates and therefore the low rates observed of type 1 diabetes are not surprising.

The 5-year cumulative incidences of autoimmune disease in our study were 5.4% and 2.2% for patients with CLE and controls, respectively. In a recent study from Taiwan, the 5-year cumulative incidences of non-SLE autoimmune disease were comparable; around 5% and 2% in patients with CLE and a matched cohort of patients with non-LE skin disease, respectively.^22^ However, there were important differences between the 2 studies: firstly, the matched cohorts were different (random sample from the general population vs patients with non-LE skin disease) and secondly, the outcome definitions for autoimmune disease differed.

In addition to a higher prevalence and incidence of autoimmune diseases, patients with CLE also had higher 5-year mortality than the controls: 10.7% vs 6.6%. This is in line with other CLE cohort studies from Denmark.^23^^,^^24^

Limitations of the present study include the risk of misclassification of patients with CLE as an unknown proportion of these patients are solely diagnosed and followed in primary care and thus not captured in the DNPR. Therefore, our study population could include fewer but potentially more severe cases, and we may have overestimated the prevalence and incidence of polyautoimmunity. Also, it would have been an advantage to the study if we had access to results of skin biopsies and blood tests. In another attempt to minimize misclassification by use of DNPR, we restricted our CLE group to patients diagnosed at dermatology departments. Furthermore, the identification of additional autoimmune diseases in the CLE group is prone to detection bias, as patients followed in the tertiary sector are possibly under higher medical surveillance. By primarily relying on DNPR for the detection of the outcome of autoimmune disease, there is a risk of missing out on diseases diagnosed and followed exclusively in primary care. Autoimmune thyroid disease is one such type of disease entity that could easily be missed, but by use of the National Prescription Register in combination with DNPR we sought to minimize the risk of outcome misclassification. Finally, there was no available information on possible confounders previously shown to be associated with development of autoimmune diseases, for example, diet, alcohol usage, physical activity, ethnicity, and particularly smoking habits.^25^, ^26^, ^27^, ^28^ A limitation to this study is the relatively homogenous population in Denmark compared to other countries, which means that our findings might not be applicable to more heterogeneous populations.

The main strength of the present study was the nationwide population-based setting. Furthermore, we were able to link the DNPR and National Prescription Register to get a more realistic estimate of the burden of autoimmune thyroid disease in these groups, and similarly, this linkage between these 2 registers likely minimized the risk of misclassification of type 1 diabetes mellitus.

In conclusion, patients with CLE have a higher prevalence and incidence of other autoimmune conditions compared with matched controls. The most prevalent autoimmune condition at time of CLE diagnosis was autoimmune thyroid disease in both patients and controls, whereas Sjogren’s syndrome and rheumatoid arthritis were the most frequent incident autoimmune diseases in the 5 years after CLE diagnosis. Clinicians should be attentive of these and other autoimmune rheumatic diseases in patients with CLE.

## Conflicts of interest

Dr Cordtz is employed at IQVIA outside of this work. Dr Dreyer has received speakers fee from Eli Lilly, Galderma, and Janssen and research grants from BMS (outside the present work). Drs Kofoed, Stensballe, Bliddal, Kristensen, Feldt-Rasmussen, and Nielsen and Authors Graven-Nielsen, Vittrup, Kragh, and Lund have no conflicts of interest to declare.

## References

[1] Grönhagen C.M., Nyberg F. (2014). Cutaneous lupus erythematosus: an update. Indian Dermatol Online J.

[2] Fairley J.L., Oon S., Saracino A.M., Nikpour M. (2020). Management of cutaneous manifestations of lupus erythematosus: a systematic review. Semin Arthritis Rheum.

[3] Petersen M.P., Möller S., Bygum A., Voss A., Bliddal M. (2018). Epidemiology of cutaneous lupus erythematosus and the associated risk of systemic lupus erythematosus: a nationwide cohort study in Denmark. Lupus.

[4] Gabriel S.E., Michaud K. (2009). Epidemiological studies in incidence, prevalence, mortality, and comorbidity of the rheumatic diseases. Arthritis Res Ther.

[5] Pons-Estel G.J., Andreoli L., Scanzi F., Cervera R., Tincani A. (2017). The antiphospholipid syndrome in patients with systemic lupus erythematosus. J Autoimmun.

[6] Bliddal S., Borresen S.W., Feldt-Rasmussen U. (2017). Thyroid autoimmunity and function after treatment with biological antirheumatic agents in rheumatoid arthritis. Front Endocrinol.

[7] Rojas-Villarraga A., Amaya-Amaya J., Rodriguez-Rodriguez A., Mantilla R.D., Anaya J.M. (2012). Introducing polyautoimmunity: secondary autoimmune diseases no longer exist. Autoimmune Dis.

[8] Matusiewicz A., Stróżyńska-Byrska J., Olesińska M. (2019). Polyautoimmunity in rheumatological conditions. Int J Rheum Dis.

[9] Kunzler E., Hynan L.S., Chong B.F. (2018). Autoimmune diseases in patients with cutaneous lupus erythematosus. JAMA Dermatol.

[10] Schmidt M., Pedersen L., Sørensen H.T. (2014). The Danish Civil Registration System as a tool in epidemiology. Eur J Epidemiol.

[11] Schmidt M., Schmidt S.A., Sandegaard J.L., Ehrenstein V., Pedersen L., Sørensen H.T. (2015). The Danish National Patient Registry: a review of content, data quality, and research potential. Clin Epidemiol.

[12] Pottegård A., Schmidt S.A.J., Wallach-Kildemoes H., Sørensen H.T., Hallas J., Schmidt M. (2017). Data Resource Profile: the Danish National Prescription Registry. Int J Epidemiol.

[13] Aalen O.O., Johansen S. (1978). An empirical transition matrix for nonhomogeneous Markov chains based on censored observations. Scand J Stat.

[14] Bliddal S., Nielsen C.H., Feldt-Rasmussen U. (2017). Recent advances in understanding autoimmune thyroid disease: the tallest tree in the forest of polyautoimmunity. F1000Res.

[15] Garelli C.J., Refat M.A., Nanaware P.P., Ramirez-Ortiz Z.G., Rashighi M., Richmond J.M. (2020). Current insights in cutaneous lupus erythematosus immunopathogenesis. Front Immunol.

[16] Klareskog L., Padyukov L., Alfredsson L. (2007). Smoking as a trigger for inflammatory rheumatic diseases. Curr Opin Rheumatol.

[17] Young K.A., Terrell D., Guthridge J. (2014). Smoking is not associated with autoantibody production in systemic lupus erythematosus patients, unaffected first-degree relatives, nor healthy controls. Lupus.

[18] Agmon-Levin N., Dagan A., Peri Y. (2017). The interaction between anti-Ro/SSA and anti-La/SSB autoantibodies and anti-infectious antibodies in a wide spectrum of auto-immune diseases: another angle of the autoimmune mosaic. Clin Exp Rheumatol.

[19] Magna M., Pisetsky D.S. (2014). The role of HMGB1 in the pathogenesis of inflammatory and autoimmune diseases. Mol Med.

[20] Paudel Y.N., Angelopoulou E., Bhuvan K.C., Piperi C., Othman I. (2019). High mobility group box 1 (HMGB1) protein in Multiple Sclerosis (MS): mechanisms and therapeutic potential. Life Sci.

[21] Shi K.Y., Kunzler E., Hynan L.S., Chong B.F. (2020). Autoimmune disease development before and after cutaneous lupus erythematosus diagnosis. Br J Dermatol.

[22] Lin T.L., Wu C.Y., Juan C.K., Chang Y.T., Chen Y.J. (2022). Long-term risk of autoimmune diseases other than systemic lupus erythematosus in cutaneous lupus erythematosus-alone patients: a 10-year nationwide cohort study. Dermatology.

[23] Hesselvig J.H., Ahlehoff O., Dreyer L., Gislason G., Kofoed K. (2017). Cutaneous lupus erythematosus and systemic lupus erythematosus are associated with clinically significant cardiovascular risk: a Danish nationwide cohort study. Lupus.

[24] Shams-Eldin A.N., Yafasova A., Faurschou M. (2022). Long-term risk of adverse cardiovascular outcomes associated with cutaneous lupus erythematosus: a nationwide cohort study. Clin Rheumatol.

[25] Carlé A., Pedersen I.B., Knudsen N. (2012). Moderate alcohol consumption may protect against overt autoimmune hypothyroidism: a population-based case-control study. Eur J Endocrinol.

[26] Crosslin K.L., Wiginton K.L. (2009). The impact of race and ethnicity on disease severity in systemic lupus erythematosus. Ethn Dis.

[27] Myles I.A. (2014). Fast food fever: reviewing the impacts of the Western diet on immunity. Nutr J.

[28] Qiu F., Liang C.L., Liu H. (2017). Impacts of cigarette smoking on immune responsiveness: up and down or upside down?. Oncotarget.

